# Association of Plasma Retinol Binding Protein-4 (RBP4) and Sonographic Grading of Fatty Liver in Obese Iranian Children

**DOI:** 10.5812/hepatmon.7103

**Published:** 2012-12-30

**Authors:** Forough Saki, Zohreh Karamizadeh, Naser Honar, Hossein Moravej, Soheil Ashkani-Esfahani, Mohammad Hossein Namvar Shooshtarian

**Affiliations:** 1 Division of Endocrinology, Department of Pediatrics, Shiraz University of Medical Sciences, Shiraz, IR Iran; 2 Deapartment of Pediatric Gastroentrology, Shiraz University of medical sciences, Shiraz, IR Iran; 3 Department of Pediatric Endocrinology, Shiraz University of medical sciences, Shiraz, IR Iran; 4 Student Research Committee, Shiraz University of Medical Sciences, Shiraz, IR Iran; 5 Department of Pathology, Ordibehesht laboratory, Shiraz, IR Iran

**Keywords:** Fatty Liver, Obesity, RBP4 Protein, Human, Child, Ultrasonography

## Abstract

**Background:**

The prevalence of obesity and its related comorbidities, such as fatty liver, in children is increasing worldwide mostly due to changes in diet and life-style. Many serological markers have been suggested for screening of fatty liver but investigations for finding more reliable factors are still in progress.

**Objectives:**

This study aimed to investigate the correlation between the level of retinol binding protein-4 (RBP4) in the serum and sonographic grading of fatty-liver severity in obese Iranian children.

**Patients and Methods:**

This case-control, double-blind study involved 51 obese children aged between five and 17 years as the case group. In addition, 35 healthy lean children with no liver problems were selected as the control group. Plasma RBP4 (using an ELISA), serum triglycerides (TG), low-density-lipoproteins (LDL), high-density-lipoproteins (HDL), total-cholesterol (Chol), and body mass index (BMI) were measured. Grading the severity of the fatty liver condition was done by an expert radiologist in the case group.

**Results:**

RBP4 levels in obese children (19 482.9 ± 3 302.2 pg/ml) were higher than those found in the lean control group (14 295.68 ± 2 381.3 pg/ml) (P < 0.05). In the obese patients, RBP4 levels showed a significant correlation with the grade of fatty liver and BMI (P < 0.05).

**Conclusions:**

It was found that the level of RBP4 had a strong correlation with the severity of fatty liver. Therefore, RBP4 may be considered as a useful, noninvasive predictive biomarker of intrahepatic lipid content in obese children prior to using radiological investigations. In particular, abdominal sonography, for the evaluation of intrahepatic lipid content in obese patients, as the sensitivity of a sonography is decreased due to the increased thickness of the abdominal wall as a result of fat deposits.

## 1. Background

The prevalence of obesity in children is increasing in many countries due to changes in diet and life-style. In fact, it is associated with a number of morbidities, such as insulin resistance, dyslipidemia, hypertension, and non-alcoholic fatty liver disease (NAFLD) (1, 2). Recently, the role of adipokines, specifically retinol binding protein-4 (RBP4) which is a transport protein for vitamin A, in the pathogenesis of obesity-related diseases, particularly NAFLD, is widely being discussed (3-6). Many studies have reported the relationship between RBP4 and obesity as well as its related complications, such as insulin resistance, metabolic syndrome, and NAFLD (5, 6). Nevertheless, there are still some discrepancies among the data from human adults. The studies conducted on pediatric patients are also of special value since they display early stages of the disease in the absence of major confounding factors, such as alcohol and other environmental influences, which are often seen in adults.

## 2. Objectives

Sonography of the liver, which is non-invasive, more practicable, and less expensive, having a sensitivity of 89% and specificity of 93% in detecting steatosis in the liver, has been found to be a good screening method for the evaluation and grading of fatty liver among different imaging and interventional diagnostic methods (7, 8). Therefore, the present study aimed to determine the correlation between plasma RBP4 levels and NAFLD as well as sonographic grading of fatty-liver in Iranian obese children between five and 17 years-of-age.

## 3. Patients and Methods

In this longitudinal, case-control, double-blind study, 51 obese children aged between 5 to 17 years (10.57 ± 3.04), who were referred to the pediatric clinics of Shiraz University of Medical Sciences between June 2011 and March 2012, were selected as the case group. In this group, 35.5% of the subjects were male and 64.5% were female (Table 1). In addition, 35 healthy, lean children (10.12 ± 3.16 years, 38% male) were matched with the case patients (P = 0.4). These children had no liver problems and were considered as the control group, they were compared with the patients in the case group in regard to RBP4 levels. The inclusion criteria for the patients were: 1) BMI > 95 percentile for their age and sex, 2) aged between 5 and 17 years, and 3) confirmation was obtained to participate in the study after explaining the study objectives in an oral presentation and signing informed consents. The exclusion criteria of the study were: 1) suffering from any other liver diseases except for NAFLD, renal failure, trauma, and acute illness, 2) using any medication (eg, oral hypoglycemic agents or hypolipidemic agents) and alcohol, and 3) suffering from diabetes mellitus. Waist circumference (WC), hip circumference (HC), and body mass index (BMI) were calculated in physical examinations by the endocrinologist examiner. Furthermore, NAFLD was diagnosed in the children according to high liver enzymes and steatosis was detected by liver sonography. Liver steatosis was graded from 0 (no steatosis) to 3 according to the Saverymuttu grading method (9). Grade 1 (mild), slightly increased echogenicity and normal visualization of the diaphragm and the intrahepatic vessels; Grade two (moderate), moderately increased echogenicity and slightly impaired visualization of the diaphragm or intrahepatic vessels; Grade three (severe), markedly increased echogenicity and poor or no visualization of the diaphragm, intrahepatic vessels, and posterior portion of the right liver lobe. Blood samples were taken from the patients at the time of the liver biopsy in order to estimate the levels of; plasma triglycerides (TG), total cholesterol (Chol), high density lipoprotein (HDL), and low density lipoprotein (LDL). Furthermore, serum RBP4 (pg/ml) was estimated by an enzyme-linked immunosorbent assay (ELISA) (Sali-Savers®, ALPCO Diagnostics, Windham, NH). The intra-assay and inter-assay coefficients of variation were 1.9–4.6% and 6.7–8.8%, respectively. The study was approved by the Ethics Committee of the Shiraz University of Medical Sciences, Shiraz, Iran.

**Table 1:** Characteristics of Study Participants, Data are Means ± SD or Percentage a

	Mean ± SD
**Age, y**	10.57 ± 3.04
**Height, cm**	141.88 ± 16.4
**Weight, kg**	62.17 ± 26.27
**BMI, kg/m^2^**	30.29 ± 8.21
**Waist circumference, cm**	87.43 ± 15.83
**Hip circumference, cm**	92 ± 15.56
**TG, mg/dl**	118.2 ± 90.9
**Chol, mg/dl**	156.47 ± 28.9
**HDL, mg/dl**	45.2 ± 10.15
**LDL, mg/dl**	86.6 ± 20
**ALT, Iu/L**	33.7 ± 44.3
**AST, Iu/L**	27.45 ± 20.27
**Alkaline phosphatase **	527.5 ± 196.8
**RBP4, pg/ml**	19 482.9 ± 3 302.2

Abbreviations: BMI, Body mass index; TG, triglycerides; Chol, cholesterol; HDL, high density lipoprotein; LDL, low density lipoprotein; ALT, alanine aminotransferase; AST, aspartate aminotransferase; RBP4, retinol binding protein

^a^These variables were log transformed before the analyses

### 3.1. Statistics

The study data were analyzed using SPSS statistical software (v. 15) (SPSS Inc., Chicago, IL, USA). Descriptive data were presented as mean ± standard deviation (SD). Comparison of RBP4 levels between the patients and the controls was done using a Student’s t-test. In addition, the correlations were evaluated by the Pearson method (10).

## 4. Results

**Figure 1:**
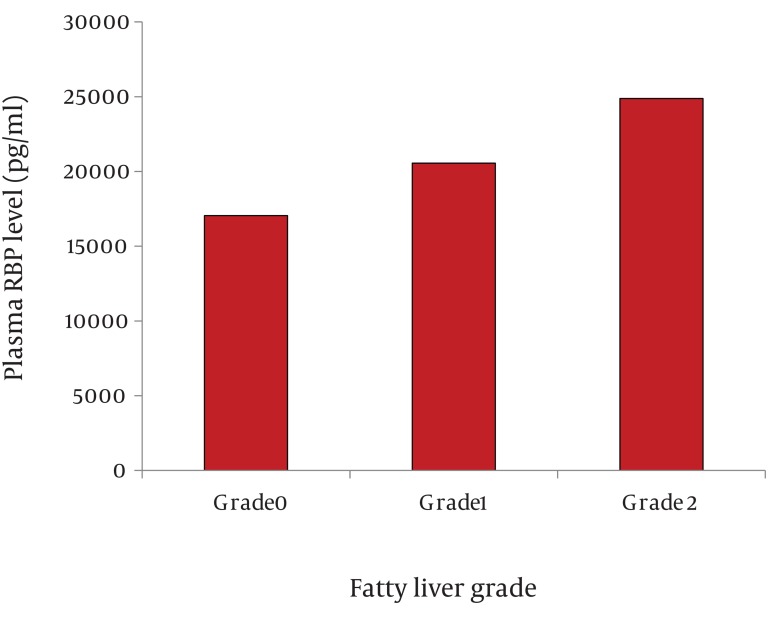
Plasma RBP4 Levels (pg/ml) According to Sonographic Grading of the Fatty Liver. A total of 51 patients were divided according to the grade of fatty liver; Grade 0 (n = 23), Grade 1 (n = 22), and Grade 2 (n = 6). The data are exhibited as mean ± SD; P < 0.05; Grade 2 vs. Grade 1 and Grade 1 vs. Grade 0.

The level of RBP4 was significantly higher in the case group (19 482.9 ± 3 302.2 pg/ml) compared to the control group (14 295.68 ± 2 381.3 pg/ml) (P < 0.001). Moreover, the level of RBP4 was 17 053.47 ± 2 199 pg/ml in obese children without sonographic evidence of fatty liver, 20 550 ± 1 990 pg/ml in grade 1 fatty liver, and 24 880 ± 1 998 pg/ml in grade 2 fatty liver (Figure 1). The greater the RBP4 level, the higher the fatty liver grade was (P < 0.001). RBP4 showed a correlation with serum TG (P = 0.002, PCI=43.6%) and BMI (P = 0.007, PCI=37.5%). However, no correlation was found between RBP4 and serum cholesterol, HDL, and LDL as well as WC and HC. Furthermore, no significant differences were observed between males and females.

## 5. Discussion

This study revealed that the level of serum RBP4, a transport protein for vitamin A, was higher in obese children than that of the lean ones. In the obese children, RBP4 showed a significant correlation with fatty liver grading. Many published studies have also indicated higher levels of plasma RBP4 in obese adults compared to the lean ones (10-15). In addition, a study conducted on type 2 diabetic adults in China showed a positive correlation between plasma RBP4 and visceral fat mass, particularly in the liver (13). Seo et al. (2008) also reported that serum RBP4 had a positive correlation with intrahepatic lipid content measured in NAFLD children (12). Moreover, Romanowska et al. (2011) performed a study on obese Polish children and demonstrated a significantly positive correlation between RBP4 and sonographic grading of fatty liver, which is in agreement with the results of the current investigation (10). However, there are some controversies among the previously published studies on the relationship between RBP4 and NAFLD. For instance, Kanaka-Gantenbein et al. reported a negative correlation between RBP4 and BMI in the study’s children, while this correlation was significantly positive in the adult participants of their study (16). On the other hand, Milner et al. (2009) displayed no differences between the controls and the NAFLD adults (17). Furthermore, Schina et al. (2009) reported even lower serum RBP4 levels among the NAFLD individuals compared to the control group (18). These findings suggest the existence of substantial physiological differences between adults and children, differences in anthropometric indices, and different methodologies of RBP4 examination and fatty liver grading. In the present investigation, RBP4 also showed a relationship with BMI as well as serum TG, which is consistent with the findings of previous studies conducted on the issue (12-17). However, no relationships were found between RBP4 and lipoproteins (HDL, LDL). Concerning the results of the previous investigations as well as those of the present study, compared to ultrasonography, RBP4 can be considered as a currently available non-invasive biomarker of intrahepatic lipid content in obese individuals, in whom the sensitivity of a sonography is decreased due to an increase of abdominal wall thickness by fat deposits. Nonetheless, these findings need to be confirmed in larger studies with biopsy proven NAFLD in order to introduce RBP4 as an alternative diagnostic method of NAFLD.
